# Effects of work-family conflict on turnover intention among primary medical staff in Huaihai Economic Zone: a mediation model through burnout

**DOI:** 10.3389/fpsyt.2023.1238315

**Published:** 2023-09-25

**Authors:** Zongliang Wen, Jintao Xu, Jinxun Yu, Xiaojing Huang, Yuting Ni

**Affiliations:** ^1^School of Management, Xuzhou Medical University, Xuzhou, China; ^2^School of Public Health, Xuzhou Medical University, Xuzhou, China; ^3^Affiliated Hospital of Xuzhou Medical University, Xuzhou, China

**Keywords:** work-family conflict, burnout, turnover intention, Huaihai Economic Zone, primary medical staff

## Abstract

**Background:**

Countries worldwide face the challenge of how medical personnel manage conflicts between work and family. Especially after the challenge of the COVID-19 epidemic, it is necessary to explore the possible mechanisms of work-family conflict, burnout, and turnover intention among primary medical staff.

**Objectives:**

This study aims to observe the turnover intention of Chinese primary medical staff and explore the relationship between work-family conflict, burnout, and turnover intention.

**Methods:**

A cross-sectional study included a turnover intention questionnaire, the Maslach Burnout Inventory-General Survey (MBI-GS), and the Work-Family Conflict Scale (WFCS) to understand turnover intention, burnout, and work-family conflict among primary medical staff in four cities (Xuzhou, Linyi, Huaibei, and Shangqiu cities) within the Huaihai Economic Zone. Spearman correlation analysis and hierarchical multiple regression analysis were used to examine the related factors of turnover intention. Structural equation modeling (SEM) was used to study the mediating role of burnout between work-family conflict and turnover intention.

**Results:**

In this study, there is a positive correlation between work-family conflict and turnover intention (*P* < 0.01). Demographic characteristics, work-family conflict, and burnout explained 2.3%, 20.3%, and 8.8% of the incremental variances, respectively. Burnout mediated the association between work-family conflict and turnover intention.

**Conclusions:**

Burnout can be regarded as a mediator between two different variables: work-family conflict and turnover intention. Improving work-family conflict and alleviating burnout may play a key role in reducing the willingness of primary medical staff to resign. Corresponding measures can be taken to balance the conflict between work and family, alleviate burnout, reduce turnover rates, and build a primary medical staff team with higher medical service quality and stability.

## 1. Introduction

Since the outbreak of COVID-19, China's grassroots medical work has become the top priority nationwide and has attracted much attention because it is the first line of defense on the anti-epidemic front. In the process of combating the epidemic, primary medical staff need to take on responsibilities beyond their original job responsibilities, such as investigating cases, providing psychological and spiritual support to patients, and facing enormous work and life pressures. They make every effort to secure medical supplies, coordinate various resources, and selflessly devote themselves to frontline pandemic prevention efforts (1, 2). After the epidemic was brought under control, China's National Health Commission also introduced a series of measures to support and strengthen primary medical institutions and staff. In addition, with the promotion of China's “Healthy China 2030” policy, primary medical staff need to take on more responsibilities, such as carrying out health education and providing basic medical services and rehabilitation services (3).

Overall, with the outbreak of the epidemic and the implementation of various health policies, primary medical work has received unprecedented attention and deeper reforms, while staff have assumed more responsibilities and obligations. Therefore, the psychological health and team stability of primary medical staff also need to be taken seriously.

Primary medical staff are a key component of the healthcare system, an important human resource of the health system, and the gatekeepers of residents' health. The role of primary medical staff in modern healthcare is becoming increasingly extensive and important (4, 5). However, due to the objective reasons of numerous service objects and high work intensity, stressors such as burnout, high work pressure, long working time, a large workload, low job satisfaction, a lack of incentive mechanisms, and low promotion prospects will adversely affect turnover intention and the stability of primary medical staff (6–9).

The motivation and tendency of in-service personnel to leave their current position is called turnover intention, which is a subjective factor that previously led to turnover behavior (10). Studies have found that turnover intention is associated with professional identity, work environment, burnout, job satisfaction, and stress (11, 12). Scholars in China and abroad have shown through research on the current situation of medical staff that there is a significant positive correlation between burnout and turnover intention, namely, the higher the level of burnout among medical staff, the higher their turnover intention (13–17). In a study involving 2,263 physicians in the United States, only 7% were certain that they would resign within 2 years (18). In Australia's primary health network, 21.3% of general practice nurses plan to leave once it is convenient, and 20.2% of general practice nurses want to leave (19).

Meanwhile, a study in the UK showed that 21.5% of primary healthcare nurses had considered quitting (20). However, the incidence of turnover intention among village doctors in China has reached 44.1%, which is twice or even six times that of primary medical staff in developed countries, seriously damaging the stability of medical staff construction and leading to the decline of medical quality (16). A systematic review and meta-analysis of the turnover intention of Chinese primary medical staff showed that, out of 37,672 primary medical staff, 30.4% are willing to resign (21). Therefore, reducing the primary medical staff's turnover intention is of great significance for stabilizing the primary medical team, heightening primary medical services' level and quality, and safeguarding people's health.

An increasing number of research literature indicates that work-family conflict can influence turnover intention in various ways, including reduction of work stress, improvements in the work environment, enhancement of the psychological contract, and an increase in work satisfaction (22–25). Work-family conflict adversely affects individuals' work and family lives to the extent that it affects overall wellbeing and health and even has dysfunctional and socially expensive effects (26). Previous research has shown that work-family conflict is an important predictive factor of turnover intention (27–29). In addition, work-family conflict could immediately or immediately affect other work-related factors such as presenteeism, anxiety, work engagement, or employee performance through mediation (30–32). In conclusion, reducing primary medical staff's work-family conflict can reduce work stress and turnover intention.

In addition to the aforementioned work-family conflict, burnout has been shown to correlate strongly with turnover intentions (33). Burnout is a long-term response to interpersonal stress and chronic negative emotions at work, defined by three dimensions: cynicism, exhaustion, and ineffectiveness (34). The relationship between turnover intention and factors related to professional identity, emotional labor, and intrinsic motivation could also be moderated by burnout (35–37). Burnout not only affects an individual's quality of life but also has an undeniable effect on organizational performance and costs (38). Burnout can lead to decreased productivity and impaired work quality.

Meanwhile, people suffering from burnout at work can affect the completion of work tasks and transmit this “work state” to colleagues, forming a “contagion” that can be sustained through social interactions at work, thereby affecting the entire work environment (26). Therefore, we hypothesized that burnout might mediate the relationship between turnover intention and other work-related factors, such as work-family conflict. Alleviating burnout among primary medical staff may play an important role in reducing their willingness to resign. Although more and more research focuses on this relationship between work-family conflict and turnover intention among primary medical staff, there is limited research on the mediating role between work-family conflict and turnover intention. Especially in China, there is currently little research on the turnover intention of primary medical staff, and most studies focus solely on the turnover intention of doctors or nurses (12, 16, 17, 22, 23, 35, 37). In rare studies on the turnover intention of primary medical staff, burnout and turnover intention have been involved, but the relationship between work-family conflict and primary medical staff turnover intention has not been explored (7, 9). Therefore, our study focused on primary medical staff in four cities (Xuzhou, Linyi, Huaibei, and Shangqiu) in the Huaihai Economic Zone to measure work-family conflict, turnover intention, and burnout and explore the relationship among them. The following hypotheses guided our analysis:

Hypothesis 1: There is no correlation between work-family conflict and turnover intention.
Hypothesis 2: Burnout has no impact on turnover intention.
Hypothesis 3: Burnout does not play a mediating role between work-family conflict and turnover intention.

## 2. Materials and methods

### 2.1. Participants, procedure, and ethics statement

On 15 March 1986, the Huaihai Economic Zone was established, with a total area of 178,000 sq. km. It has 14 prefecture-level cities in Jiangsu, Anhui, Henan, and Shandong provinces and is one of the earliest regional economic cooperation organizations in China. The Huaihai Economic Zone is the intersection of these four provinces. The development speed of cities in this region is relatively slow, with a weak economic foundation and less support from higher-level governments. However, with a large population of 120 million, the task of providing grassroots medical care is significant. Moreover, the cultural and social customs in the region are similar, and the research has promotional significance.

From February 2023 to March 2023, according to the distribution of population and the distribution of primary medical staff, a cross-sectional survey with a simple random sampling design was conducted in Xuzhou, Linyi, Huaibei, and Shangqiu cities of China through multiple communications with the staff of local health commission departments.

The survey was distributed by the administrative personnel of the local Municipal Health Commission to all primary medical institutions within their jurisdiction. Before the survey, all primary medical staff learned about the study through the WeChat working group, knew the purpose, risks, benefits, and process of the study, and ensured the confidentiality of the data. Then, they chose whether to participate in the questionnaire according to their own situation. The questionnaire, developed by Wenjuanxing—a widely used online survey platform in China–was distributed to respondents' mobile phones either as QR codes or links to the questionnaire website. After the respondents submitted their informed consent form, they filled out the corresponding questionnaire on their mobile phones. If there was a pattern of inconsistent answers in the respondents' questionnaire filling (such as selecting the same option for the entire questionnaire) or incomplete filling, the response was considered invalid and was deleted. Respondents had to be at least 20 years old, should have worked for more than 6 months, be limited to doctors, nurses, pharmacists, or administrators, and should voluntarily participate in the survey. The informed consent form included the study's purpose, risks, benefits, and process. In particular, strict protection of their privacy should be ensured in this study.

The questionnaire contained a turnover intention questionnaire, the Maslach Burnout Inventory-General Survey (MBI-GS), the Work-Family Conflict Scale (WFCS), and self-developed questions on demographic characteristics. The validated questionnaire took ~20 min to complete. A total of 2,501 survey questionnaires were received, and 2,259 valid questionnaires were collected, with an effective recovery rate of 90.32%. The study complied with the Declaration of Helsinki as revised in 1989, and the protocol was authorized by the Ethics Committee of Xuzhou Medical University (ID: 225281).

### 2.2. Baseline characteristics of the primary medical staff

We collected baseline characteristics of primary medical staff, which include age, gender (men and women), monthly income [≤ 3,000 yuan (≤ US $430.9), 3,001–5,000 yuan (US $430.9–$718.2) and >5,000 yuan (>US$718.2)], education (junior college and below, bachelor degree and above), specialty (clinical medicine, medical technology, preventive medicine, nursing, pharmacy or else), technical title (no title, primary title, middle title, vice-senior title, or above), years of work (<2, 3–5, 6–9, or ≥10 years), and weekly hours at work (≤ 40, >40).

### 2.3. Assessment of turnover intention

We used the Chinese version of the turnover intention questionnaire developed by Cammannet and Mobiley to measure respondents' turnover intention (39). It consists of four items: “Thought of leaving this industry,” “Thought of leaving the organization you serve now,” “Looking for a new job next year,” and “Looking for a new job recently.” The Likert scale uses a 5-point scale, ranging from 1 (very dissatisfied) to 5 (very satisfied), to evaluate all of these items, where higher scores indicate a higher willingness to resign. The Cronbach's alpha coefficient for turnover intention in this study was 0.883.

### 2.4. Assessment of work-family conflict

The Chinese version of the multidimensional Work-Family Conflict Scale (WFCS) was adopted to evaluate work-family conflict (40). WFC consists of 18 items with two subscales: work-to-family interference (WIF) and family-to-work interference (FIW). WIF refers to the negative impact of work-related requirements and obligations on family life. FIW means that conflicts caused by family obligations can interfere with personal work. Both subscales include conflicts of time, stress, and behavior. Work-family conflict was evaluated using a 5-point Likert scale, ranging from 1 (highly disagree) to 5 (highly agree). The Cronbach's alpha for WFC in this study was 0.940.

### 2.5. Assessment of burnout

The burnout of primary medical staff was evaluated with the Chinese version of the Maslach Burnout Inventory-General Survey (MBI-GS) (33, 34). The scale includes three subscales: emotional exhaustion, depersonalization, and low personal achievement (reverse score). The response was set to a 7-Likert score, ranging from 0 (never) to 6 (daily). Among them, emotional exhaustion scores >25, depersonalization scores >11, and low personal achievement scores >16 were considered to be high burnout. Burnout can be diagnosed when the score of any dimension of the respondents is greater than the critical value. The Cronbach's alpha coefficient for MBI-GS in this study was 0.793.

### 2.6. Statistical analyses

Statistical analysis was conducted using SPSS version 23.0 and Amos 26.0 Windows statistical software (IBM Corporation). The Kruskal–Wallis test and one-way analysis of variance (ANOVA) were used to explore the relationship between turnover intention and demographic variables. Pearson correlation analysis was used to examine the correlation between turnover intention, burnout, and work-family conflict.

We used hierarchical multiple regression (HMR) analysis methods to explore the relevant factors affecting turnover intention: Step 1: baseline characteristics of the primary medical staff; Step 2: work-family conflict of the primary medical staff; and Step 3: burnout of the primary medical staff. As a dependent variable, the turnover intention score is continuous in HMR. Using standardized parameter estimation (standardized β) to evaluate the degree of correlation between the dependent and independent variables. We used structural equation modeling (SEM) to explore the mediating role of burnout, with turnover intention as the dependent variable, work-family conflict as the independent variable, and burnout as the mediating variable. A two-tailed *P*-value of <0.05 was considered statistically significant.

## 3. Results

### 3.1. Baseline characteristics of the participants

The baseline characteristics and distribution of turnover intention among primary medical staff are shown in Table 1. A total of 2,259 primary medical staff members participated in the study. Nearly 90% of the participants were over 30 years old (2,022, 89.51%), and more than 60% of the participants were women (1,431, 63.35%). Over three-quarters of the participants had a junior college degree or below (1,709, 75.65%). The number of participants with a primary title is the highest (1,017, 44.98%), and the primary title is the most basic level of technical title. Nearly three-quarters of the participants had worked for over 10 years (1,685, 74.59%). The results of univariate analysis showed that participants over 30 years old had lower turnover intention scores than participants under 30 years old (*P* = 0.044). Female participants had lower turnover intention (*P* = 0.005). The higher the monthly income of participants, the lower the turnover intention score (*P* = 0.019). At the same time, the turnover intention scores were different among participants with different specialties (*P* < 0.001). Participants with a higher technical title had lower turnover intention (*P* = 0.018).

**Table 1:** Baseline characteristics of the distribution of the turnover intention among primary medical staff (*N* = 2,259).

**Variable**	***N* (%)**	**Turnover intention (mean ±SD)**	***P***
**Age (years)**			0.044
≤ 30	237 (10.49)	8.07 ± 3.74	
>30	2,022 (89.51)	7.57 ± 3.64	
**Gender**			0.005
Men	828 (36.65)	7.99 ± 3.95	
Women	1,431 (63.35)	7.41 ± 3.46	
**Education**			0.235
Junior college and below	1,709 (75.65)	7.61 ± 3.73	
Bachelor's degree and above	550 (24.35)	7.66 ± 3.43	
**Monthly income (**¥^a^**)**			0.019
<3,000	1,295 (57.33)	7.85 ± 3.82	
3,000–5,000	625 (27.67)	7.40 ± 3.49	
>5,000	339 (15.01)	7.16 ± 3.25	
**Specialty**			<0.001
Clinical medicine	1,140 (50.46)	7.92 ± 3.73	
Medical technology	111 (4.91)	7.37 ± 3.49	
Preventive medicine	236 (10.45)	7.22 ± 3.70	
Nursing	475 (21.03)	7.62 ± 3.51	
Pharmacy	106 (4.69)	6.13 ± 2.89	
Else	191 (8.46)	7.28 ± 3.76	
**Technical title**			0.018
No title	727 (32.18)	7.58 ± 3.76	
Primary title	1,016 (44.98)	7.81 ± 3.74	
Middle title	380 (16.82)	7.53 ± 3.45	
Vice-senior title and above	136 (6.02)	6.65 ± 2.82	
**Years of work**			0.482
<2	137 (6.06)	7.15 ± 3.58	
3–5	193 (8.54)	7.58 ± 3.51	
6–9	244 (10.80)	7.69 ± 3.72	
≥10	1,685 (74.59)	7.65 ± 3.67	

^a^1¥ = US $0.14.

P: The possibility the data could have occurred under the null hypothesis. Bold values indicates the *p* value was less than 0.05, which had statistical significance.

### 3.2. Turnover intention of the participants

The average score of the primary medical staff's turnover intention was 7.62 ± 3.66. The total score of turnover intention is less than half of the total score (out of 20), and the level of turnover intention among primary medical staff is relatively low. The item with the highest average score is “Thought of leaving the organization you serve now,” with an average score of 2.20 ± 1.17. The item with the lowest average score is “Looking for a new job next year,” with an average score of 1.62 ± 0.93.

### 3.3. Work-family conflict among the participants

The average score of the primary medical staff's work-family conflict was 45.68 ± 14.47. The total score of work-family conflict is higher than half of the total score (out of 90), and the level of work-family conflict among primary medical staff is relatively high. The highest average score is the impact of time in WIF, with an average score of 9.52 ± 3.39. The lowest average score is the impact of stress in FIW, with an average score of 5.82 ± 2.81. The average total score of WIF is 8.52 ± 3.34. The average total score of FIW is 6.70 ± 3.03.

### 3.4. Burnout of the participants

The average score of the primary medical staff's burnout was 36.00 ± 14.32. The total burnout score is less than half of the total score (out of 132), and the level of burnout among primary medical staff is low. The average scores of emotional exhaustion, depersonalization, and low personal achievement were 12.37 ± 7.72, 2.88 ± 3.22, and 20.75 ± 9.18 in this study, respectively.

### 3.5. Correlations between turnover intention, work-family conflict, and burnout

Table 2 shows that there was a significant correlation between work-family conflict, burnout, and turnover intention (*P* < 0.01). Work-family conflict had a positive correlation with turnover intention (*P* < 0.01). At the same time, burnout had a positive correlation with both work-family conflict and turnover intention (*P* < 0.01).

**Table 2:** The correlations among turnover intention, work-family conflict, and burnout.

	**1**	**2**	**3**
1. Turnover intention	1		
2. Work-family conflict	0.462^***^	1	
3. Burnout	0.474^***^	0.446^***^	1

^***^Significant at the 0.01 level (two-tailed).

### 3.6. Factors associated with turnover intention

Table 3 shows the final results of the HMR model for the turnover intention of primary medical staff. The final model explained a total variance of 31.3%. Demographic characteristics, work-family conflict, and burnout demonstrated 2.3%, 20.3%, and 8.8% of the incremental variance, respectively. According to Table 4, age was negatively correlated with turnover intention (*P* = 0.050). Meanwhile, higher monthly income was negatively correlated with turnover intention (*P* = 0.005).

**Table 3:** The hierarchical multiple regression analysis of turnover intention of the primary medical staff.

**Variable**	**Model 1**			**Model 2**			**Model 3**		
	β	**Standardized** β	**95% CI**	β	**Standardized** β	**95%CI**	β	**Standardized** β	**95% CI**
**Demographic characteristics**									
Age (years)	−1.168^**^	−0.098^**^	−1.815 to −0.522	−0.998^**^	−0.084^**^	−1.574 to −0.423	−0.801^**^	−0.067^**^	−1.344 to −0.259
Gender	−0.433^*^	−0.057^*^	−0.770 to −0.095	−0.044	−0.006	−0.346 to 0.258	−0.124	−0.016	−0.408 to 0.160
Education	0.495^*^	0.058^*^	0.097 to 0.892	0.375^*^	0.044^*^	0.021 to 0.729	0.364^*^	0.043^*^	0.031 to 0.698
Monthly income (¥^a^)	−0.431^**^	−0.087^**^	−0.685 to −0.176	−0.326^**^	−0.066^**^	−0.552 to −0.099	−0.339^**^	−0.068^**^	−0.553 to −0.126
Specialty	−0.119^*^	−0.056^*^	−0.214 to −0.025	−0.028	−0.013	−0.113 to 0.056	−0.049	−0.023	−0.129 to 0.030
Technical title	0.072	0.017	−0.147 to 0.291	0.107	0.025	−0.088 to 0.302	0.113	0.027	−0.070 to 0.297
Years of work	0.340^**^	0.082^**^	0.105 to 0.574	0.085	0.021	−0.125 to 0.295	0.089	0.021	−0.109 to 0.286
Work-family conflict				0.117^**^	0.463^**^	0.107 to 0.126	0.078^**^	0.310^**^	0.068 to 0.088
Burnout							0.085^**^	0.332^**^	0.075 to 0.095
*R* ^2^		**0.023**			**0.226**			**0.313**	
Adjusted *R*^2^		**0.020**			**0.223**			**0.311**	
Δ*R*^2^		**0.023**			**0.203**			**0.088**	

^*^P <0.05.

^**^P <0.01.

^a^1¥ = US $0.14.

Model 1 contains the following variables: age, gender, education, monthly income, specialty, technical title, and years of work.

Model 2 contains the following variables: age, gender, education, monthly income, specialty, technical title, years of work, and work-family conflict.

Model 3 contains the following variables: age, gender, education, monthly income, specialty, technical title, years of work, work-family conflict, and burnout.

R^2^: A statistical measure in a regression model that determines the proportion of variance.

Adjusted R^2^: A modified version of R^2^ that accounts for predictors that are not significant in a regression model.

ΔR^2^: The change in R^2^ between two equations. Bold values highlight the statistical measure in a regression model for a more intuitive and clear comparison.

**Table 4:** The correlations among turnover intention with baseline characteristics.

**Variable**	**Correlation**	***P***
Age (years)	−0.041^*^	**0.050**
Gender	−0.060^**^	**0.004**
Education	0.025	0.235
Monthly income (¥^a^)	−0.059^**^	**0.005**
Specialty	−0.086^**^	**<0.001**
Technical title	0.010	0.643
Years of work	0.020	0.344

^*^P <0.05.

^**^P <0.01. Bold values indicates the *p* value was less than 0.05, which had statistical significance. ^*a*^1¥ = US $0.14.

### 3.7. Burnout as a mediator between work-family conflict and turnover intention

**Figure 1:**
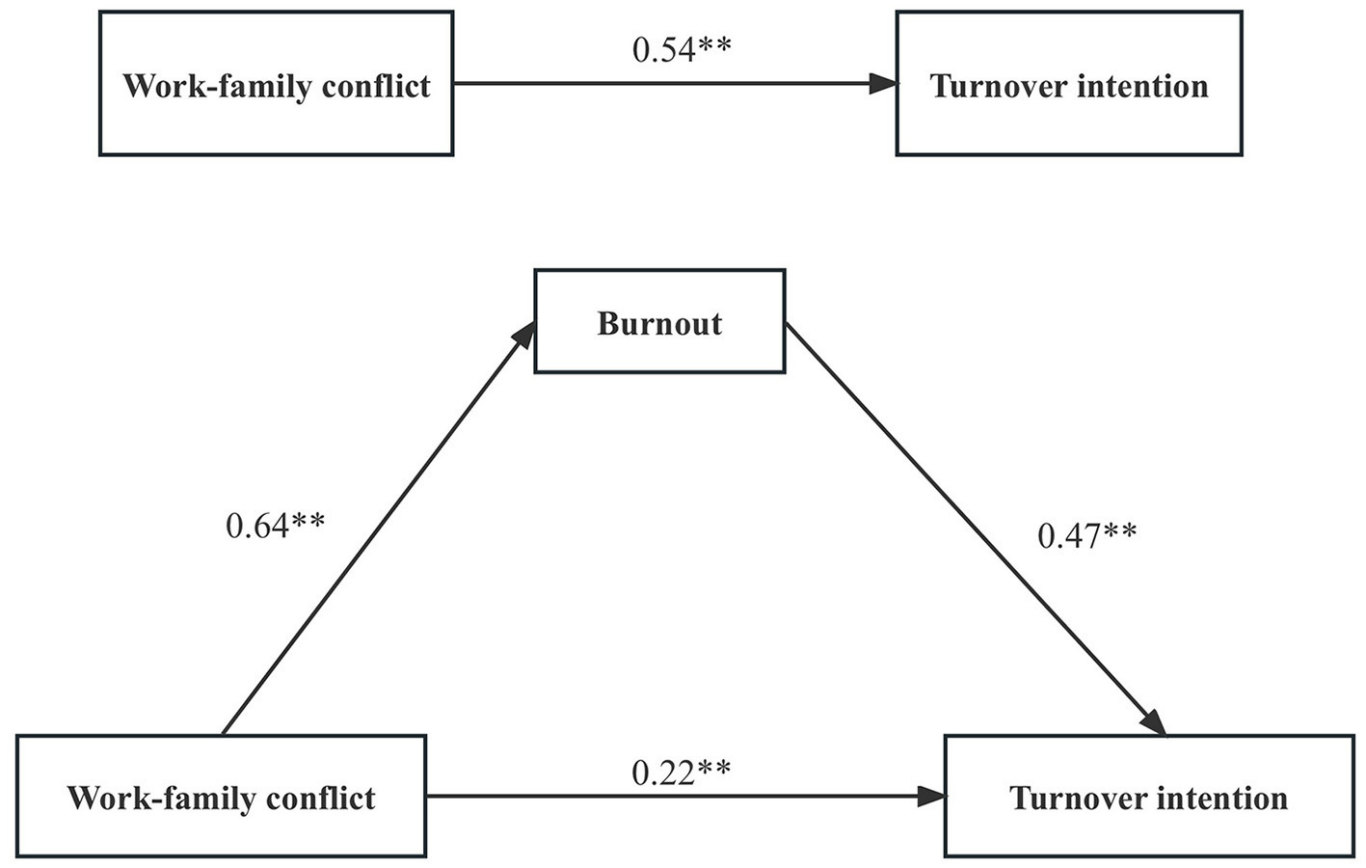
Standardized solution for the structural equation model of work-family conflict and turnover intention. Standardized solution for the structural equation model of burnout, work-family conflict, and turnover intention. **P < 0.01.

Table 5 shows the results of the SEM analysis. The path coefficients and standardized solutions of SEM are shown in Figure 1. Work-family conflict influenced turnover intention directly (*c* = 0.54, *P* < 0.001). Work-family conflict had a positive correlation with turnover intention (*P* < 0.001). The model fitting indices meet the requirements (χ^2^/df = 1.763 <5, GFI = 0.996 > 0.9, AGFI = 0.992 > 0.9, CFI = 0.999 > 0.9, TLI = 0.998 > 0.9, RMSEA = 0.018 <0.05; Figure 1). Burnout was significantly correlated with turnover intention (*P* < 0.001).

**Table 5:** The path coefficients of the mediation model.

	***B***	**β**	**S.E**.	**C.R**.	***P***
Burnout ← work-family conflict	0.257	0.642	0.010	25.987	<0.001
Turnover intention ← burnout	0.613	0.470	0.047	12.976	<0.001
Turnover intention ← work-family conflict	0.114	0.217	0.016	7.048	<0.001

B, the unstandardized path coefficient; β, the standardized path coefficient; S.E., the standard error; C.R., the critical ratio.

When burnout served as a mediator, the path coefficient between work-family conflict and turnover intention significantly decreased (*c* = 0.22, *P* < 0.001; Figure 1). After adding burnout mediation, the direct path coefficient of work-family conflict on turnover intention decreased or had no statistical significance, which also indicated the existence of a mediating effect. Burnout mediated the association between work-family conflict and turnover intention (a^*^b = 0.30, BCa 95% CI: 0.249–0.355, Percentile 95% CI: 0.260–0.360). The model showed a good fit across multiple indices: (χ^2^/df = 2.922 <5, GFI = 0.991 > 0.9, AGFI = 0.982 > 0.9, CFI = 0.995 > 0.9, TLI = 0.992 > 0.9, and RMSEA = 0.029 <0.05).

## 4. Discussion

This study aims to examine whether the primary medical staff's turnover intention is related to burnout and work-family conflict and further explore whether burnout plays a mediating role in work-family conflict and turnover intention. The study indicates significant correlations among turnover intention, work-family conflict, and burnout. In addition, this study observed that burnout, to some extent, mediates the relationship between work-family conflict and turnover intention, and the mediating effect is significant. Although there had been studies exploring the relationship between turnover intention, burnout, and work-family conflict, there was limited research literature on the three factors mentioned above among primary medical staff, and there was even more limited research on the role of burnout in the relationship between work-family conflict and turnover intention (41–45). Therefore, attention needs to be paid to work-family conflict, burnout, and turnover intention among primary medical staff (46–48).

This study found that work-family conflict was positively correlated with turnover intention, which is consistent with previous research studies (49, 50). Primary medical staff have a close relationship with the general public and fully understand the local people's family environment, living conditions, and disease history. To provide comprehensive and continuous health services to residents and ensure the health needs of urban and rural residents, they undertook special and complex work tasks, which are not only heavy workloads but also very trivial (51). The huge workload will inevitably lead to the physical and mental exhaustion of primary medical staff, and returning to the family in such a state will inevitably cause issues when handling family affairs. Through HMR analysis, this study found that work-family conflict is a risk factor for turnover intention, indicating that an increase in work-family conflict may lead to higher turnover intention. For married female doctors, factors such as work-family conflict and psychological empowerment were particularly relevant to turnover intention (52). Moreover, when studying the relevant factors of nurses' turnover intention, it was also observed that work-family conflict could predict turnover intention (53). Therefore, attention should be paid to the harm of work to individuals and families and the support of families for work. In addition, the perception of work-family conflict could hinder individuals from entering the field or increase the likelihood of providers leaving the field, exacerbating healthcare shortages (54, 55). Consequently, we should take proactive and effective measures to maintain a balance between work and family, thereby reducing turnover intention among primary medical staff.

Burnout is a long-term response to interpersonal stress and chronic emotions at work (34). When there is a conflict between work and family, and primary medical staff are unable to balance this conflict, it may lead to burnout in this job. Therefore, this study found a positive correlation between work-family conflict and burnout. Previous studies have found that reducing work-family conflict can alleviate the negative consequences of burnout, such as reduced anxiety symptoms, improved professional efficacy and emotional exhaustion, and reduced work pressure (56–58). In practice, reducing the work pressure on primary medical staff can effectively reduce burnout. Studies have found that work pressure affects burnout through work-family conflicts and anxiety symptoms among female nurses (22, 59). The above research indicates that work-family conflict can lead to a decline in an individual's emotional wellbeing, cognitive investment, and overall engagement in the workplace, thereby enhancing the turnover intention. Many scholars have noticed the negative impact of burnout, but few studies have focused on the role of burnout in the relationship between work-family conflict and turnover intention. Therefore, this study provides new inspiration for revealing how work-family conflict affects primary medical staff's careers and turnover intention.

In terms of outcomes, burnout is often associated with various forms of negative reactions and job withdrawal, including absenteeism, low organizational commitment, job dissatisfaction, intention to leave, and turnover (42, 60–63). Consistent with previous studies, this study observed a positive correlation between burnout and turnover intention (60, 61). Burnout often manifests as a negative and negligent approach to work, underestimating one's own work value, and being indifferent to others. Meanwhile, burnout can affect the entire work environment through social interactions at work (34). HMR analysis showed that burnout is a risk factor for high turnover intention. In practice, when there is a high level of work-family conflict, primary medical staff may experience burnout from their work. The work-family conflict itself can lead to a desire to resign, and long-term burnout can exacerbate the desire to resign. Therefore, burnout played a mediating role between work-family conflict and turnover intention. When burnout is considered a mediator, it can buffer the impact of work-family conflicts on turnover intention, indicating that it can effectively control and reduce the conflict between work and family and reduce the willingness to resign. The quality and stability of the staff of primary medical institutions are directly related to the quality and effectiveness of primary medical services and are also closely related to the success or failure of the medical service system reform. Under such high job standards, burnout was a serious factor that affected the work environment and could exacerbate the willingness of primary medical staff to resign (62–64).

Therefore, according to our research results, work-family conflict and burnout are key factors affecting the turnover intention of primary medical staff, and burnout is a mediator between work-family conflict and turnover intention. Thus, we should take effective measures to reduce work-family conflict, burnout, and turnover intention. By creating a positive and harmonious work environment, we can reduce burnout, improve the work satisfaction of primary medical staff, further reduce their willingness to resign, improve the internal stability of the organization, achieve a win-win situation for the organization and primary medical staff, and ultimately improve the quality of medical services.

## 5. Limitations

Several limitations should be acknowledged in this study. First, since the survey was conducted among primary medical staff in four cities in China–Xuzhou, Linyi, Huaibei, and Shangqiu—the generalizability to other populations is limited. Second, since this study was carried out from February 2023 to March 2023, the outcomes of this study may be limited by the COVID-19 pandemic and other unmeasured confounders. Third, selection bias cannot be ruled out because the survey was conducted online, and the participants were smartphone users only. Finally, this study did not differentiate among service items. Due to a lack of health workforce, respondents generally undertake multiple items. Future research may find differences when investigating this issue within certain primary medical staff profiles.

## 6. Conclusions

The research results confirm that work-family conflict and burnout have a direct positive impact on turnover intention, and burnout plays a mediating role between work-family conflict and turnover intention. Therefore, the study draws the following conclusion: first, work-family conflict is a risk factor for turnover intention, which can directly lead to turnover intention. Second, burnout is a risk factor for turnover intention and has a positive effect on turnover intention. Finally, work-family conflict, turnover intention, and burnout can form a mediated effect model, and burnout as a mediation variable can increase the impact of work-family conflict on turnover intention. This study has scientific and practical significance for reducing the talent turnover rate in the primary medical system. Primary medical institutions are the foundation of China's health system. Low employee retention rates may lead to a decline in the quality of diagnosis and treatment and the level of medical services, seriously affecting the stability and sustainability of primary healthcare and thus posing a potential threat to public health. In this context, effective psychological intervention measures should be taken to improve the psychological quality and stress resistance of primary medical staff so that they can better adapt to the high intensity and pressure of primary medical work, reduce work-family conflict and burnout, protect the psychological health of primary medical staff, reduce their willingness to resign, and improve medical quality and service level.

## Data availability statement

The raw data supporting the conclusions of this article will be made available by the authors, without undue reservation.

## Ethics statement

The studies involving human participants were reviewed and approved by Ethics Committee of Xuzhou Medical University. Written informed consent for participation was obtained.

## Author contributions

ZW and JX designed the study, conducted the literature review, and wrote the research protocol. JX and JY contributed to the acquisition and interpretation of data and drafted and revised the manuscript. JX, XH, and YN contributed to the revisions in depth for the manuscript. All authors contributed to the article and approved the final manuscript.
