# Supplementary material for: Effects of work-family conflict on turnover intention among primary medical staff in Huaihai Economic Zone: a mediation model through burnout
Source: Front Psychiatry. 2023 Sep 25;14:1238315. doi: 10.3389/fpsyt.2023.1238315 (PMC10561281; doi:10.3389/fpsyt.2023.1238315)
Supplement: Supplementary file 1 [file Table_1.DOCX]

Supplementary Material

**Questionnaire on work-family conflict, burnout and turnover intention of primary medical staff**

Dear primary medical staff,

Hello! In order to understand the current situation and problems of primary medical staff in primary medical institutions, we designed a questionnaire to investigate. The questionnaire is anonymous. You do not have to worry, we will keep the content of your answer confidential. Please fill in all the items in the questionnaire truthfully. We would like to express our heartfelt thanks to you for your support and help.

Please select Province City and region: [Fill in the blanks] *

_________________________________

Hospital name [fill in the blank] *

_________________________________

Your gender: [Single choicee] *

| ○Male | ○Female |  |  |  |  |  |  |
| --- | --- | --- | --- | --- | --- | --- | --- |

Your age: [Single choice]*

| ○Age 30and under |  |  |  |
| --- | --- | --- | --- |
| ○Over 30 years old |  |  |  |

Your education level: [Single choice] *

| ○ Technical Secondary School |
| --- |
| ○ Junior College |
| ○ Undergraduate |
| ○ Master's Degree |
| ○ Doctor |

Your speciality: [Single choice] *

| ○ Clinical Medicine |
| --- |
| ○ Medical Technology |
| ○ Preventive medicine |
| ○ Nursing |
| ○ Pharmacy |
| ○ else |

Your technical title: [Single choice] *

| ○No title  ○Primary title  ○Middle title  ○Vice-senior title and above |
| --- |

Your average monthly income [Single choice] *

| ○3000 RMB and below |
| --- |
| ○3001RMB~5000RMB |
| ○8001 RMB and above |

Your years of work [Single choice] *

| ○2 years and below |
| --- |
| ○3-5 years |
| ○6-9 years |
| ○10 years and above |

Based on your feelings and experiences over the past month, please answer the following questions and choose the most suitable one for your personal situation. [Matrix Single Choice Question] *

|  | never | seldom | occasionally | often | frequently | very frequently | daily |
| --- | --- | --- | --- | --- | --- | --- | --- |
| C1.Work sometimes brings me down | ○ | ○ | ○ | ○ | ○ | ○ | ○ |
| C2.I feel very tired after a day's work | ○ | ○ | ○ | ○ | ○ | ○ | ○ |
| C3.I wake up in the morning feeling very tired, but still have to face the work of the day | ○ | ○ | ○ | ○ | ○ | ○ | ○ |
| C4.I can easily understand how patients feel about things | ○ | ○ | ○ | ○ | ○ | ○ | ○ |
| C5.Sometimes patients are treated as objects rather than as human beings | ○ | ○ | ○ | ○ | ○ | ○ | ○ |
| C6.Working with people all day is a burden to me | ○ | ○ | ○ | ○ | ○ | ○ | ○ |
| C7.I am able to deal with patients' problems effectively | ○ | ○ | ○ | ○ | ○ | ○ | ○ |
| C8.My work exhausts me | ○ | ○ | ○ | ○ | ○ | ○ | ○ |
| C9.I feel that my work makes a positive difference in the lives of others | ○ | ○ | ○ | ○ | ○ | ○ | ○ |
| C10.Since taking this job, I have become more and more indifferent to people | ○ | ○ | ○ | ○ | ○ | ○ | ○ |
| C11.I'm afraid this job will make me a hardened person | ○ | ○ | ○ | ○ | ○ | ○ | ○ |
| Normal people have five fingers on one hand, please select the “daily” option. |  |  |  |  |  |  |  |
| C12.I feel energetic | ○ | ○ | ○ | ○ | ○ | ○ | ○ |
| C13.I feel frustrated in my work | ○ | ○ | ○ | ○ | ○ | ○ | ○ |
| C14.I feel that my work is too hard | ○ | ○ | ○ | ○ | ○ | ○ | ○ |
| C15.I don't care much about the needs of my patients | ○ | ○ | ○ | ○ | ○ | ○ | ○ |
| C16.Working with other people makes me feel a lot of pressure | ○ | ○ | ○ | ○ | ○ | ○ | ○ |
| C17.When I'm with patients, I can easily create a relaxed atmosphere | ○ | ○ | ○ | ○ | ○ | ○ | ○ |
| C18.The close contact with patients in my work makes me feel satisfied and happy | ○ | ○ | ○ | ○ | ○ | ○ | ○ |
| C19.Working in health care has made me experience my own value | ○ | ○ | ○ | ○ | ○ | ○ | ○ |
| C20.I felt myself at the end of my rope | ○ | ○ | ○ | ○ | ○ | ○ | ○ |
| C21.I can calmly deal with the emotional troubles I encounter at work | ○ | ○ | ○ | ○ | ○ | ○ | ○ |
| C22.I think some patients blame me for their problems | ○ | ○ | ○ | ○ | ○ | ○ | ○ |

Work-Family Conflict Survey

The title of this section is about the description of work-family conflict. Based on your actual work situation, please choose the one that fits you between the 5 levels of "highly disagree" and "highly agree". [Matrix Single Choice Question] *

|  | highly disagree | Less disagree | normal | Less agree | highly agree |
| --- | --- | --- | --- | --- | --- |
| D1. My job often prevents me from attending family events | ○ | ○ | ○ | ○ | ○ |
| D2. I spend so much time at work that I don't have enough time for family activities | ○ | ○ | ○ | ○ | ○ |
| D3. I have to sacrifice time for family activities to complete necessary work tasks | ○ | ○ | ○ | ○ | ○ |
| D4. The time I spend on my family obligations usually interferes with the completion of my work | ○ | ○ | ○ | ○ | ○ |
| D5. Spending time with my family prevented me from participating in work activities that would benefit my career | ○ | ○ | ○ | ○ | ○ |
| D6. I have to miss work activities because I have to spend a lot of time fulfilling my family responsibilities | ○ | ○ | ○ | ○ | ○ |
| D7. When I come home from work, I am often too tired to meet my family obligations | ○ | ○ | ○ | ○ | ○ |
| D8. When I come home from work, I am often depressed and unable to contribute to the family | ○ | ○ | ○ | ○ | ○ |
| D9. Because of the pressure at work, sometimes when I come home I can't do the things I like because of the pressure | ○ | ○ | ○ | ○ | ○ |
| Is it true that 1+1=2? Please select the “highly agree” option |  |  |  |  |  |
| D10. Due to the great pressure of my family, I often focus on my family at work | ○ | ○ | ○ | ○ | ○ |
| D11. It is difficult for me to concentrate on my work due to the pressure brought on by family responsibilities | ○ | ○ | ○ | ○ | ○ |
| D12. Stress and anxiety from home life often reduce my ability to work | ○ | ○ | ○ | ○ | ○ |
| D13. The methods used to solve problems at work are not effective in solving problems at home | ○ | ○ | ○ | ○ | ○ |
| D14. What is necessary and effective at work is counterproductive at home | ○ | ○ | ○ | ○ | ○ |
| D15. Being effective at doing things at work doesn't make me a good parent or partner | ○ | ○ | ○ | ○ | ○ |
| D16. The methods used to solve problems at home are not effective in solving problems at work | ○ | ○ | ○ | ○ | ○ |
| D17. What is necessary and effective at home is counterproductive at work | ○ | ○ | ○ | ○ | ○ |
| D18. Behaviors that help solve problems at home don't seem to be effective at work | ○ | ○ | ○ | ○ | ○ |

According to the actual situation of your work, choose one of the five grades from "very dissatisfied" to "very satisfied". [Matrix Single Choice Question] *

|  | very dissatisfied | relative disagreement | normal | relative agreement | very satisfied |
| --- | --- | --- | --- | --- | --- |
| E1. I thought about leaving my current employer | ○ | ○ | ○ | ○ | ○ |
| E2. Thought about leaving the industry | ○ | ○ | ○ | ○ | ○ |
| E3. Recently looking for a new job | ○ | ○ | ○ | ○ | ○ |
| E4. I will be looking for a new job next year | ○ | ○ | ○ | ○ | ○ |
